# Annual of the Universal Medical Sciences

**Published:** 1890-09

**Authors:** 


ANNUAL OF THE UNIVERSAL MEDICAL SCIENCES. A Yearly Report of the Progress of the General Sanitary Sciences throughout the World. Edited by CHARLES E. SAJOUS, M. D., and Seventy Associate Editors, assisted by over Two Hundred Corresponding Editors, Collaborators and Correspondents. Illustrated with chromo-lithographs, engravings and maps. Five volumes. Philadelphia: F. A. Davis. 1890.

This valuable reference work has again appeared with promptitude, and with even greater completeness than before. The labor is so enormous and the difficulties so many to be encountered in the preparation of a work of such vastness, the great wonder is that the subscribers are able to obtain its benefits as early in the year as they do.

Some improvements have been added to this year's issue. These consist of the creation of new departments, as well as the modification of others. The newly-created departments are: Syphilis, edited by Professor William J. White, Philadelphia; Surgical Mycoses, edited by Prof. Ernest Laplace, of Philadelphia; and Thoracic Surgery, edited by Professor J. McFadden Gaston, of Atlanta.

The sections of Oral Surgery, Orthopedic Surgery, Bacteriology, and Therapeutics have undergone modifications, while all the improvements of last year have been continued. The publisher still maintains the high order of excellence with which he began his work, while the engravers are entitled to much praise for the generally good quality of the illustrations. This annual is fast becoming a necessity for the medical writer and teacher, as well as for the practitioner, and it will not be long before the wonder will be expressed on all sides that such a convenience was not put forth years before Dr. Sajous volunteered to do so.

We have heretofore commended the enterprise of the editor in the conception of and carrying forward to perfection this great literary scheme, and we still desire to add such encouragement as good words may afford towards a continuation of the Annual on the lines already cast.

